# Emulsion templated scaffolds with tunable mechanical properties for bone tissue engineering

**DOI:** 10.1016/j.jmbbm.2015.09.019

**Published:** 2016-02

**Authors:** Robert Owen, Colin Sherborne, Thomas Paterson, Nicola H. Green, Gwendolen C. Reilly, Frederik Claeyssens

**Affiliations:** a Department of Materials Science and Engineering, University of Sheffield, INSIGNEO Institute for in silico medicine, The Pam Liversidge Building, Sir Frederick Mappin Building, Mappin Street, Sheffield S1 3JD, United Kingdom; b Department of Materials Science and Engineering, University of Sheffield, The Kroto Research Institute, North Campus, Broad Lane, Sheffield S3 7HQ, United Kingdom

**Keywords:** Porosity, Free form fabrication, Mechanical properties, PolyHIPEs, Plasma polymerization, Stereolithography

## Abstract

Polymerised High Internal Phase Emulsions (PolyHIPEs) are manufactured via emulsion templating and exhibit a highly interconnected microporosity. These materials are commonly used as thin membranes for 3D cell culture. This study uses emulsion templating in combination with microstereolithography to fabricate PolyHIPE scaffolds with a tightly controlled and reproducible architecture. This combination of methods produces hierarchical structures, where the microstructural properties can be independently controlled from the scaffold macrostructure. PolyHIPEs were fabricated with varying ratios of two acrylate monomers (2-ethylhexyl acrylate (EHA) and isobornyl acrylate (IBOA)) and varying nominal porosity to tune mechanical properties. Young’s modulus, ultimate tensile stress (UTS) and elongation at failure were determined for twenty EHA/IBOA compositions. Moduli ranged from 63.01±9.13 to 0.36±0.04 MPa, UTS from 2.03±0.33 to 0.11±0.01 MPa and failure strain from 21.86±2.87% to 2.60±0.61%. Selected compositions were fabricated into macro-porous woodpile structures, plasma treated with air or acrylic acid and seeded with human embryonic stem-cell derived mesenchymal progenitor cells (hES-MPs). Confocal and two-photon microscopy confirmed cell proliferation and penetration into the micro- and macro-porous architecture. The scaffolds supported osteogenic differentiation of mesenchymal cells and interestingly, the stiffest IBOA-based scaffolds that were plasma treated with acrylic acid promoted osteogenesis more strongly than the other scaffolds.

## 1 Introduction

3-D tissue scaffold manufacturing processes are divided into techniques that produce 3-D objects with random microstructure via stochastic processes (e.g. electrospinning, high internal phase emulsions, solvent casting and particulate leaching) (Hutmacher, 2000); and additive manufacturing techniques that produce structures with ordered user-controlled microstructure (e.g. stereolithography, fused deposition modelling and selected laser sintering) via computer aided design/manufacturing (CAD/CAM) (Hutmacher, 2001). Random networks, when compared to user-defined structures, are generally easier to produce in bulk but their microstructure and physical properties are more difficult to control, analyse and interpret. Control over pore distribution and interconnectivity can be achieved using additive manufacturing techniques, which are ideally suited for exploring the relationship between 3-D topography and cell attachment, proliferation and differentiation. This requires optimising the geometrical parameters of the scaffold, i.e. the dimensions of the scaffold fibres vs. the pores (Hollister, 2005, Danilevicius et al., 2015). The fibres ensure the mechanical stability of the scaffold, while the pores allow for mass transport to aid cell/nutrient delivery and tissue generation. High resolution and throughput techniques such as microstereolithography permit the fabrication of scaffolds that have a highly controlled architecture with well-defined pores and interconnectivity, improving cell growth and tissue formation (Lee et al., 2011).

Additive manufacture and emulsion templating have been previously combined to produce structures with multi-scale porosity (Cooperstein et al., 2015, Sušec et al., 2013, Johnson et al., 2013). Materials formed by emulsion templating two immiscible liquids (termed the internal phase and continuous phase) with the internal phase volume ratio (*ϕ*) exceeding 0.7405, are termed HIPEs. For the majority of photocurable HIPEs, the internal phase is water and the continuous phase is a hydrophobic liquid formed from the monomer(s), crosslinker, surfactant, and photoinitiator (Cameron and Sherrington, 1996, Cameron, 2005). Polymerisation of the continuous phase results in a PolyHIPE; a permeable, highly interconnected, porous network with a low bulk density where the percentage porosity is simply *ϕ* (Caldwell et al., 2012). We previously showed that a hierarchical porosity can be introduced into scaffolds using microstereolithography through the use of a High Internal Phase Emulsion (HIPE) as a substrate material (Johnson et al., 2013). This hybrid biomaterial manufacturing technique enables the hierarchical structuring of scaffolds where the microstructure is controlled by emulsion templating while the macrostructure is governed by additive manufacturing.

Acrylate-based PolyHIPEs with specific mechanical properties can be fabricated using different monomers. By altering the proportions of the elastomer 2-ethylhexyl acrylate (EHA) and the monomer isobornyl acrylate (IBOA), different stiffness can be obtained (Johnson et al., 2013, Jerenec et al., 2014). The ability to control the mechanical properties of PolyHIPEs allows scaffolds to be tuned depending on the application. For example, flexible scaffolds are needed for mechanical conditioning of cells in compression; however, scaffolds need to be stiff enough to fill a load-bearing defect and to be handled during surgery. In addition, it has been demonstrated that substrate stiffness, nanotopography and binding site spacing all effect stem cell differentiation and matrix production (Engler et al., 2006, Trappmann et al., 2012, Evans et al., 2009, Viswanathan et al., 2015, Stevens and George, 2005).

The ease with which porous materials can be fabricated using emulsion templating makes PolyHIPEs excellent materials for 3-D cell culture, as exemplified by the commercialisation of Alvetex^®,^ a polystyrene-based PolyHIPE scaffold (Knight et al., 2011). Cellular penetration into these PolyHIPE monoliths is dependent on their thickness and pore size (Hayward et al., 2013a); however, Akay et al. (2004) found that regardless of pore size, cellular penetration in PolyHIPEs was rarely seen beyond 1 mm. Additionally, when plasma treating the PolyHIPE to overcome the intrinsic hydrophobicity, it has been shown that there is a significant depth dependence with regards to its efficiency. Plasma treatments have been shown to coat the inner-surfaces of an 85% porous, 10 mm diameter 3 mm thick disc; however, any porous object beyond a few millimetres thick will not be homogenously coated, with the least deposition occurring at the core (Barry et al., 2005, Viswanathan et al., 2014). Therefore, PolyHIPE monoliths need to be thin for optimal cell and plasma penetration. By creating scaffolds from HIPEs using microstereolithography, this depth limit can be overcome as individual fibres will not be too thick for cell ingrowth and plasma penetration (<1 mm), but the overall depth of the scaffold can be much larger than for monoliths. This can have many applications, especially in bone repair, such as a for synthetic bone graft substitutes or as a scaffold for cell-based bone regeneration. In addition, macro-pores can be created in the scaffold that are large enough to permit vascularisation, whereas the smaller micro-pores present on a PolyHIPE monolith would not. In general, cells need to be within 200 μm of a blood vessel to receive oxygen and nutrients, and for sufficient vascularisation of a scaffold, a pore size of at least 300 μm has been recommended (Cao et al., 2006).

Current bone graft substitutes, usually ceramic based, have had some success but are brittle and difficult to shape and therefore are usually provided as granules (Hing et al., 2007, Hing, 2005). Bone tissue engineering is a promising alternative that could overcome many of these complications, but it is yet to proceed to mainstream clinical practise due to limitations such as the need to retain both mechanical strength and adequate porosity for sufficient and timely vascularisation of scaffolds after implantation (Amini and Laurencin, 2012).

Our aim in this study was to synthesise a range of PolyHIPEs with tunable mechanical properties to support bone cells in vitro. PolyHIPEs of different stiffness were selected and used to manufacture hierarchically structured 3-D scaffolds for cell culture using a woodpile design. Scaffolds were seeded with human embryonic stem-cell derived mesenchymal progenitors (hES-MPs), and their ability to support attachment, proliferation and osteogenic differentiation was assessed.

## 2 Materials and methods

### 2.1 HIPE synthesis

HIPEs were synthesised with monomer proportions ranging from 100% EHA (Sigma Aldrich, UK) to 100% IBOA (Sigma Aldrich, UK) at 25% intervals. The organic component of the continuous phase was formed from the monomers and a crosslinker (trimethylolpropane triacrylate (TMPTA) Sigma Aldrich, UK) at 26.96 wt% of the monomers. A surfactant (Hypermer B246-SO-(MV), Croda, UK) was added at 3 wt% of the organic mass and left to dissolve in a sonic water bath. Finally, a photoinitiator (2,4,6-trimethylbenzoyl)-phosphine oxide/2-hydroxy-2-methylpropiophenone, 50/50, Sigma Aldrich, UK) was added at 5 wt% of the organic mass. The internal phase, distilled water, was added at 0.75, 0.80, 0.85 and 0.90 *ϕ* to each continuous phase, to produce twenty HIPE compositions. These are referred to by their wt% EHA and nominal porosity. For example, EHA50P85 is a HIPE consisting of 50 wt% EHA and 50 wt% IBOA with a *ϕ* of 0.85, and EHA0P75 is a HIPE formed from 100% IBOA with a *ϕ* of 0.75.

### 2.2 Fabrication of PolyHIPE sheets for tensile testing

Sheets of PolyHIPE were fabricated from each composition and laser-cut to size based on ASTM D638-10, the standard test method for tensile properties of plastics (American Society for Testing and Materials, 2010). HIPE was pipetted into a silicone mould and cured to form a sheet using an automated UV belt curer (GEW Mini Laboratory, GEW engineering UV), washed in acetone and dried overnight. The size of the tensile samples was scaled down from the dimensions stated in ASTM D638-10 due to the maximum sample height that could be tested in the extensometer. Therefore, longitudinal dimensions were reduced by a factor of 3.83, but original axial dimensions were not altered to retain the cross-sectional area. Samples were cut using a laser cutter (Mini 18 Laser, Epilog Laser) with an intensity of 8%, speed of 70% and a frequency of 2500 Hz. The number of passes required was dependent on the porosity and thickness of the PolyHIPE.

### 2.3 Mechanical characterisation of PolyHIPE tensile samples

Samples were tested on a BOSE ElectroForce 3200 mechanical testing machine using a 450 N load cell, an extension rate of 0.02 mm/s, a grip distance of 10 mm, and a maximum extension of 6 mm. Each composition was tested and the Young’s modulus (E), ultimate tensile stress (UTS) and percentage elongation at failure determined. The UTS was calculated as the maximum force applied divided by the sample cross sectional area, and percentage elongation at failure expressed as the extension at failure divided by the original distance between the grips (10 mm for all samples). The Young׳s modulus of each sample was determined using the gradient of the linear-elastic region of the force–displacement curve. For all samples, the initial point from which this was measured was at an extension of 0.02 mm, and the final point taken was at yield. Compositions selected for cell culture were also tested after soaking in PBS for 1 h to investigate whether the stiffness was affected by the sample being wet.

### 2.4 Physical characterisation of PolyHIPE tensile samples

Scanning electron microscopy (SEM) was used to examine how changes in composition affected the degree of openness (DOO) of the PolyHIPE (Pulko and Krajnc, 2012). For each composition, a sample was mounted on a carbon tab; sputter coated with gold (SC500, emscope) at a pressure of 0.05 atm and a current of 15 mA for two minutes, and then imaged using a Philips XL-20 SEM with an electron beam with energy of 20 kV. Images at 400× magnification were analysed using the measurement tool in Image J (Schneider et al., 2012).

DOO is the ratio of open surfaces (*S_o_*) to the total surface of a cavity (*S*_*c*_). *S*_*c*_ is calculated using the measured void diameter (*D*_*m*_) multiplied by a statistical correction factor to represent the equatorial void diameter (*D_e_*) (Eq. (1)) (Carnachan et al., May 2006)

(1) $$S_{c}=\pi {\left(\frac{2D_{m}}{\sqrt{3}}\right)}^{2}$$

To determine *S_o_*, the diameters of the visible interconnects were averaged (*D_i_*) and used to calculate the average area ($\pi {(\frac{D_{i}}{2})}^{2}$). This was multiplied by the number of visible interconnects (*N*), then by 2 as the void had been cut in half, and by the statistical correction factor as it is not known where the cavity had been bisected. The DOO is simply the ratio of *S_o_* to *S_c_* (Eq. (2))

(2) $$\mathrm{Degree}\;\mathrm{of}\;\mathrm{Openness}\;\left(DOO\right)=\frac{\mathrm{Open}\;\mathrm{Surface}\;\mathrm{of}\;\mathrm{Cavity}}{\mathrm{Total}\;\mathrm{Surface}\;\mathrm{of}\;\mathrm{Cavity}}=\frac{N*2*\frac{2}{\sqrt{3}}*\pi {\left(\frac{D_{i}}{2}\right)}^{2}}{\pi {D_{e}}^{2}}$$

### 2.5 Fabrication of PolyHIPE scaffolds

Scaffolds were fabricated onto 13 mm glass coverslips. To functionalise the coverslips so that the polymer adhered, they were treated with piranha solution (80 vol% H_2_SO_4_ (Sigma Aldrich, UK), 20 vol% H_2_O_2_ (Sigma Aldrich, UK)), washed in distilled water, methanol-dried, and added to a solution of 10 wt% 3-methylacryloxypropyltrimethoxysilane (MAPTMS, Polysciences Inc.) in toluene. Before use, coverslips were washed in methanol and dried.

Four layer woodpile scaffolds were fabricated from EHA0P80, EHA50P80 and EHA100P80 PolyHIPEs using a single-photon direct-write microstereolithography setup. A subnanosecond pulse duration, passively Q-switched DPSS microchip laser (PULSELAS-P355-300, ALPHALAS, Germany), controlled using a laser diode and thermoelectric cooler driver (LDD1-1BT-D, ALPHALAS, Germany), emitting wavelengths of 1064, 532 and 355 nm was used as a source. A Pellin–Broca prism (ADB-10, THORLABS, UK) was used to separate a single wavelength of 355 nm. Beam delivery was controlled with a shutter (UNIBLITZ LS6, VincentAssociates, Canada) linked to a shutter driver (VCM-D1, VincentAssociates, Canada) and intensity with a pinhole. The beam was focused through a microscope objective (EC-Plan NEOFLUAR 10×, Carl Zeiss Ltd, UK), and a high precision stage with the ability to move in all three planes (ANT130-XY (Aerotech, UK) for *xy* translation & PRO115 (Aerotech, UK) for *z* translation) commanded by a motion controller and software (A3200 Software-Based Machine Controlled (Aerotech, UK)) was used to translate the focal spot. The laser was focused just above the coverslip-HIPE interface for the bottom layer and the fibre-HIPE interface for each subsequent layer in order to write the scaffold.

**Fig. 1:**
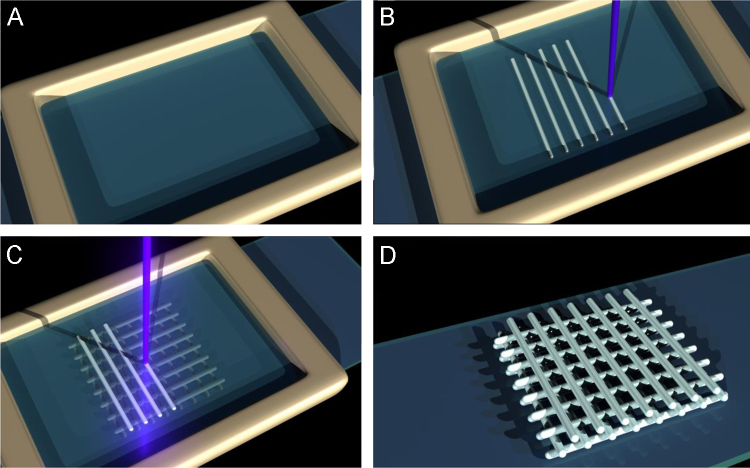
Scaffold manufacture process. (A) HIPE is pipetted onto a functionalised coverslip (B) Laser beam is focused onto the coverslip-HIPE interface and writes the bottom layers. These fibres attach to the coverslip (C) Additional HIPE added covering the first layer of fibres. Laser beam focussed to fibre-HIPE interface and second layer is written. These fibres attach to the first fibre layer. This is repeated until scaffold is complete (D) After fabrication excess polymer is removed and the scaffold is washed in acetone before drying.

For all compositions, a current of 2.20 μA and a pinhole size of 3.1 mm was used, resulting in a measured laser power of 1.5 mW on the sample (measured by a SC310 thermal power sensor and a PM50 controller, THORLABS, UK). However, for each of the different compositions it was necessary to tune the process parameters (e.g. the volume of HIPE used and write speed) to address differences in curing properties and to ensured that the macrostructure of each scaffold was similar between compositions. To write the scaffold, a layer of HIPE was pipetted onto a functionalised coverslip, placed onto the stage and the first layer fabricated. Additional HIPE was added after the completion of each layer. Once completed, scaffolds were washed with acetone and dried with a heat gun. For this study, in total 336 scaffolds were fabricated, each taking approximately 13 min to produce (Fig. 1).

### 2.6 Plasma modification of scaffolds

The continuous phase of a HIPE typically utilises a hydrophobic monomer in order to form the emulsion. Therefore, to promote cell adhesion, spreading and proliferation, the surface chemistry of the produced scaffold was altered via plasma modification using either an air plasma clean (pcAir) or air plasma followed by plasma deposited acrylic acid (pdAAc).

Treatments were applied by placing the scaffolds centrally in a cylindrical plasma chamber with stainless steel endplates, wrapped with a coil of wire connected to a 13.56 MHz frequency generator. For pcAir scaffolds, the pressure was adjusted to 1.8×10^−1^ mbar and the power set to 50 W to generate the plasma. Samples were exposed to the plasma for 5 min. For pdAAc scaffolds, samples were kept in the chamber after exposure to air plasma and liquid nitrogen added to the cold trap. Once the pressure dropped to 3.0×10^−3^ mbar, a flask of acrylic acid (Sigma Aldrich, UK) was attached to the inlet and the pressure adjusted to 3.0×10^−2^ mbar. Acrylic acid plasma was then generated for 10 min using a power of 15 W and a flow rate of 2.40–2.50 sccm^−1^. If samples were not used immediately, they were kept under vacuum until needed.

### 2.7 Cell culture

hES-MPs (Cellartis, Sweden), mesenchymal progenitors, were used to assess the suitability of the PolyHIPE scaffolds to support osteogenic precursors. Cells were cultured at 37 °C, 5% CO_2_ in basal media (BM), containing Minimum Essential Alpha Medium (α-MEM, Lonza, UK), 10% foetal bovine serum (FBS, Labtech, UK), 2 mM L-glutamine (Sigma Aldrich, UK) and 100 mg/mL penicillin/streptomycin (Sigma Aldrich, UK) and in gelatine-coated T75 flasks. BM was supplemented with human fibroblastic growth factor (Life Technologies, UK) at 4 ng/ml and media was changed every 2–3 days.

Cells were used between the third and sixth passage, and depending on the experiment, cultured in either osteogenesis induction media (OIM) or supplemented media (SM). OIM is BM supplemented with ascorbic acid (50 µg/mL (Sigma Aldrich, UK)), beta-glycerolphosphate (βGP, 5 mM (Sigma Aldrich, UK)) and dexamethasone (100 nM (Sigma Aldrich, UK)). SM is the same composition as OIM but without dexamethasone.

Scaffolds were sterilised by soaking in 70 vol% ethanol for 2 h before being washed three times in sterile PBS. Scaffolds were seeded with 75,000 cells at a density of 1,500,000 cells/mL in a non-treated 24 well plate, to ensure cell attachment only occurred on the scaffold, and left for 45 min to attach. 1 mL of BM was then added to each well to submerge the scaffolds and incubated overnight. On day 1, scaffolds were transferred to a 12 well plate and 2 mL of OIM or SM added to selected scaffolds. Media was changed every 2–3 days.

#### 2.7.1 Assessment of the suitability of acrylate-based PolyHIPEs for bone tissue engineering

To assess the suitability of the PolyHIPE scaffolds for cell culture, resazurin reduction (RR) assays were performed. Resazurin solution is reduced to resorufin by the cells, changing the colour of the media from a non-fluorescent blue to a highly fluorescent pink. The fluorescence is measured using a microplate reader and is correlated with cell viability (O׳Brien et al., 2000).

The RR assay was performed at three time points (Day 1, 8, 15). 1 mM Resazurin Sodium Salt (Sigma Aldrich, UK) in dH_2_O was diluted in BM (10 vol%) to create a RR solution. Culture media was removed from the samples and replaced with 2 ml of RR solution, the well plates were wrapped in aluminium foil and incubated for 4 h at 37 °C. 200 µl of the reduced solution was added to a 96-well plate and measured using a spectrofluorometer (FL_X_800, BIO-TEK Instruments, Inc.) at an excitation wavelength of 540 nm and an emission wavelength of 630 nm. Scaffolds were washed twice with PBS before adding fresh media.

#### 2.7.2 Evaluation of the effects of PolyHIPE composition on alkaline phosphatase activity

The activity of ALP, an enzyme involved in the bone mineralisation process, can be used as an early indicator of osteogenic differentiation (Farley et al., 1983, Hoemann et al., 2009). ALP activity was measured on days 8 and 15 on pdAAc and pcAir scaffolds cultured in OIM and SM. Culture media was removed from the scaffolds, they were washed twice in PBS and 500 µl of cell digestion buffer (10 v/v% cell assay buffer (1.5 M Tris–HCl, 1 mM ZnCl_2_, 1 mM MgCl_2_ in deionised water (diH_2_O), 1% Triton-X100 (Sigma Aldrich, UK), in diH_2_O) was added to each scaffold and incubated for 30 min. Scaffolds were then removed and the lysates transferred to 1.5 mL tubes, vortexed briefly, then stored overnight at 4 °C. The lysates then underwent a freeze-thaw cycle three times (−80 °C 10 min, 37 °C 15 min), before being vortexed for 15 seconds per sample. Finally, they were centrifuged at 10,000 rpm for 5 min.

10 µl of the lysate was combined with 190 µl of PNPP Phosphatase Substrate (Thermo Scientific, UK) and added to a 96-well plate, then incubated at 37 °C until a slight colour change from colourless to yellow was observed. Absorbance was then measured using a plate reader (EL_X_800, BIO-TEK) at a wavelength of 405 nm every minute for 30 min. ALP activity is expressed as nmol of *p*-nitrophenol per minute (nmol pNP/min), assuming that one absorbance value equals 22.5 nmol of product.

#### 2.7.3 PicoGreen^®^ assay

A Quant-iT™ PicoGreen^®^ dsDNA Assay (PG) Kit (Life Technologies, UK) was used to determine the amount of double stranded DNA (dsDNA) present in the cell lysate, indicating cell number. PG reagent is a flurochrome which binds with dsDNA. When excited, the fluorescence value correlates to the amount of dsDNA present in the sample (Ahn et al., 1996). 180 µl of the lysate was mixed in a 1:1 ratio with the PG working solution (1:20 Tris-EDTA (TE) buffer (10 mM Tris–HCl, 1 mM EDTA, pH 7.5), 1:200 PG reagent in dH_2_O) in a 1.5 mL tube. This mixed solution was transferred to an opaque well plate, wrapped in foil and incubated at room temperature for 10 min. Samples were then read using a spectrofluorometer at an excitation wavelength of 485 nm and an emission wavelength of 528 nm.

#### 2.7.4 Immunolabelling, confocal, and two-photon microscopy

The scaffolds with the highest levels of proliferation, as determined from day 15 RR assay results, were imaged using confocal microscopy to view cell location on the scaffold. Samples were stained with DAPI (4′-, 6-diamidino-2-phenylindole dihydrochloride (Sigma Aldrich, UK) and Phalloidin-TRITC (Phalloidin–Tetramethylrhodamine B isothiocyanate (Sigma Aldrich, UK)) in order to view nuclei and f-actin, respectively. Two-photon microscopy was used to assess cell penetration into the scaffold pores using the same staining protocol.

To stain the cells, the media was removed and the scaffolds washed twice with PBS. They were then fixed with 1 ml of 3.7% formaldehyde (Sigma Aldrich, UK) and left for 20 min before being washed with PBS a further 2 times. 1 ml of immunocytochemistry (ICC) buffer (1% BSA, 0.1% Triton-X100 in PBS) was then added and left for 20 min before removing the buffer, adding 500 µl of phalloidin working solution (1:1000 phalloidin stock solution (1 mg phalloidin-TRITC, 1.5 ml methanol) in ICC buffer), and wrapping the well plate in foil. After 30 min, the staining solution was removed and the scaffold washed twice with PBS, then 1 ml of DAPI working solution (0.1 vol% DAPI in PBS) was added and left wrapped in foil for 10 min. The DAPI working solution was then removed and the scaffolds washed once in PBS. Samples were submerged in PBS, wrapped in foil, and refrigerated until use.

Confocal images (512×512 pixels) were obtained using an upright microscope (Axioskop 2 FS MOT Microscope, Carl Zeiss Ltd, UK) with a 10× objective (W N-Achroplan 10×/0.3, Carl Zeiss Ltd, UK) and a pixel dwell time of 2.56 µs. DAPI was detected using a tunable Ti-Sapphire two-photon laser (*λ_ex_* 800 nm, *λ_em_* 435–485 nm) and Phalloidin-TRITC detected using a single photon laser (*λ_ex_* 543 nm, *λ_em_* 565–615 nm). 3-D images of the scaffolds were produced using ‘*z*-stacking’, with between 25 and 100 images, taken at 8.74 µm intervals. Differential interference contrast (DIC) images were also taken at the middle slice of the z-stack. This shows the position of the scaffold fibres without any cells, which when viewed side by side with the confocal images, allows for the plane of the image within the scaffold to be easily determined.

Two-photon images (512×512 pixels) were obtained using the upright microscope with a 40× objective (Achroplan 40×/0.75 W, Carl Zeiss Ltd, UK) and a pixel dwell time of 6.39 μs. Both fluorophores and the PolyHIPE material were detected using a tunable Ti-Sapphire two-photon laser (*λ_ex_* 800 nm), with the signal detected as follows: DAPI – *λ_em_* 435–485 nm, Phalloidin-TRITC – *λ_em_* 565–661 nm, and PolyHIPE autofluorescence – *λ_em_*>560 nm. Images were taken at 1 µm intervals to create a *Z*-stack.

#### 2.7.5 Statistical analysis

All mechanical analysis was performed with ten samples at each composition. If samples slipped or broke in the grips, the results for that sample were discarded. When calculating DOO, ten voids were selected randomly for measurement from an SEM image of each composition. Cell viability experiments were repeated three times in triplicate. Outliers were removed using the ROUT method (*Q*=5%). ALP and PicoGreen experiments were repeated twice in triplicate. Two-way ANOVA with Tukey׳s post-test was used to evaluate significant differences, all graphs are presented at mean±SD and notable significant differences are indicated on the graphs or in the legends.

## 3 Results

### 3.1 Mechanical characterisation of PolyHIPE tensile samples

**Fig. 2:**
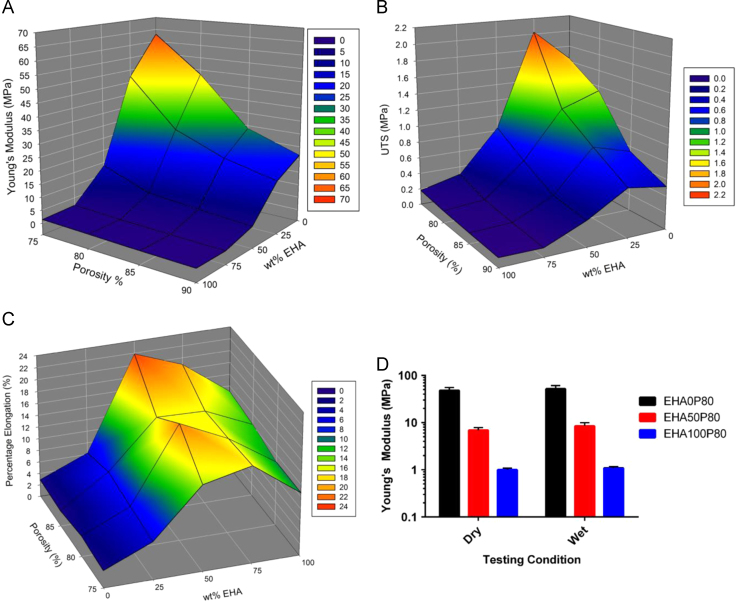
Surface plots showing the effects of nominal porosity and wt% EHA on (A) Young’s modulus, (B) Ultimate Tensile Stress, (C) percentage elongation at failure (D) mean±SD of the dry and wet stiffness for the compositions used for cell culture. For A–C, the nodes indicate the mean of ten samples. There were no significant differences for dry vs. wet at any of the compositions.

PolyHIPE sheets had measured stiffness in tension ranging from 63.01±9.13 MPa to 0.36±0.04 MPa, with the highest Young’s moduli occurring in the EHA0P75 composition and the lowest at EHA100P90, as expected (Fig. 2A). Both higher porosity and a higher wt% of EHA resulted in a lower stiffness. However, the monomer composition had the greatest influence.

As with Young’s modulus, an increase in *ϕ* at the same wt% EHA causes a reduction in UTS (Fig. 2B). However, the highest UTS was achieved by EHA25P75 (2.03±0.33 MPa), rather than EHA0P75 (1.64±0.22 MPa). The highest UTS at 0.75, 0.85 and 0.90 *ϕ* is achieved by an EHA25 composition , although at all four nominal porosities the difference between the EHA0 and EHA25 UTSs is not significant (*p*<0.05).

The percentage elongation at failure is not affected by the nominal porosity at each composition (Fig. 2C). It is only influenced by the monomer compositions with the ductility increasing with the addition of EHA, peaking around the EHA50 and EHA75 compositions and then declining. As EHA is an elastomer, this is expected.

Tensile samples for each composition were not always made from the same batch of polymer, yet there was a high degree of concordance for the stress–strain curves, indicating that the synthesis method can reproducibly create the same material. Testing of wet samples showed that cell culture conditions had no effect on the stiffness of the material (Fig. 2D).

### 3.2 Degree of openness

**Fig. 3:**
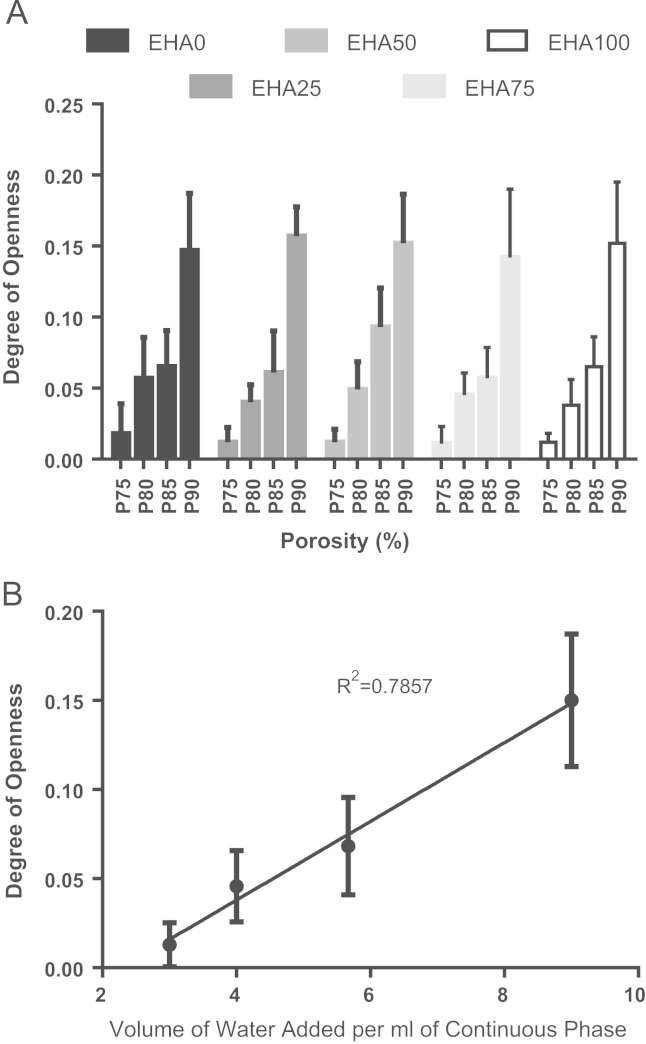
(A) Mean±SD of the DOO for each of the 20 compositions. (B) Average DOO vs. nominal porosity expressed as volume of water added per 1 mL of continuous phase. Nominal porosities combined for compositions. R2 calculated using linear regression, slope is significantly non-zero (*p*<0.0001).

To determine whether the physical effects of the different nominal porosities were the same for each composition, DOO values were compared. In all cases, a higher DOO was observed at higher porosities (Fig. 3A). At a given porosity, there was no significant difference in DOO between monomer proportions for 0.75, 0.80 and 0.90 *ϕ* (*p*<0.05), and only EHA25P85 vs. EHA50P85 and EHA50P85 vs. EHA75P85 were significantly different (*p*<0.05). For each composition, the largest increase in the DOO was observed when *ϕ* increased from 0.75 to 0.80 and 0.85 to 0.90, with a relatively smaller difference between 0.80 and 0.85. The low DOO at 0.75 *ϕ* is because the emulsion is only classed as a HIPE at 0.7405 *ϕ*. If nominal porosity is viewed as the volume of water added per 1ml of continuous phase to achieve this *ϕ*, a linear relationship can be seen (Fig. 3B). This is because the porosity is reciprocal to the amount of polymer.

### 3.3 Scaffold fabrication

**Fig. 4:**
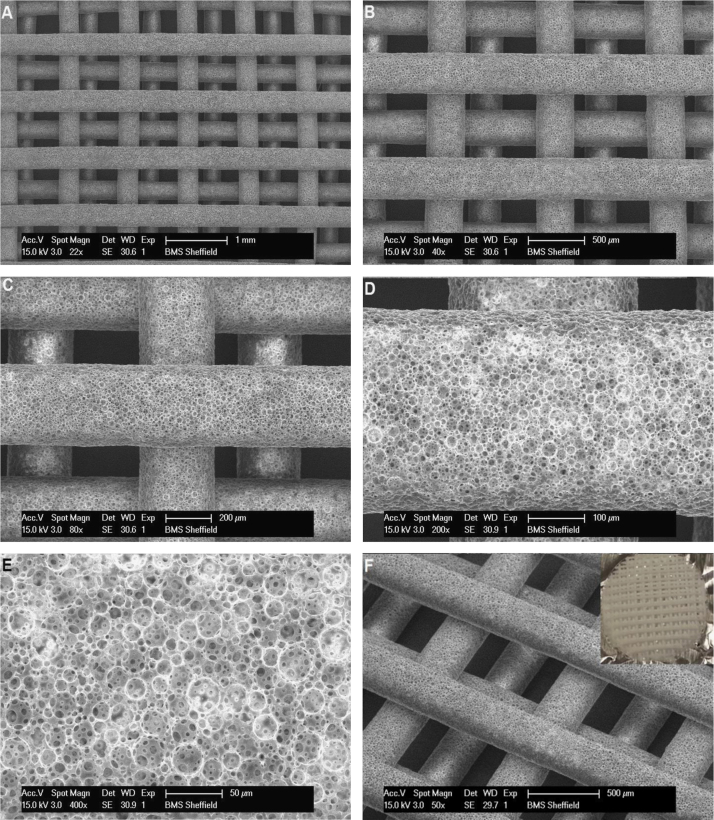
SEM images of an EHA0P80 4 layer woodpile scaffold. (A–E) Magnification of the same point from 22× to 400×, showing the inherent macroscopic and microscopic porosity of the structure. (F – Main) A side view of one of the fibres showing the offset of the upper two layers. Scale bars A–F: 1 mm, 500 μm, 200 μm, 100 μm, 50 μm, 500 μm. (F-Insert) Photograph of a single scaffold.

Scaffolds were reproducibly fabricated from the three compositions (EHA0P80, EHA50P80, EHA100P80), with a fibre diameter and spacing of approximately 350 µm and 650 µm, respectively (Fig. 4). The third and fourth layers were offset so that the top layers lay directly above the gaps of the previous layer.

### 3.4 Cell metabolic activity assay

**Fig. 5:**
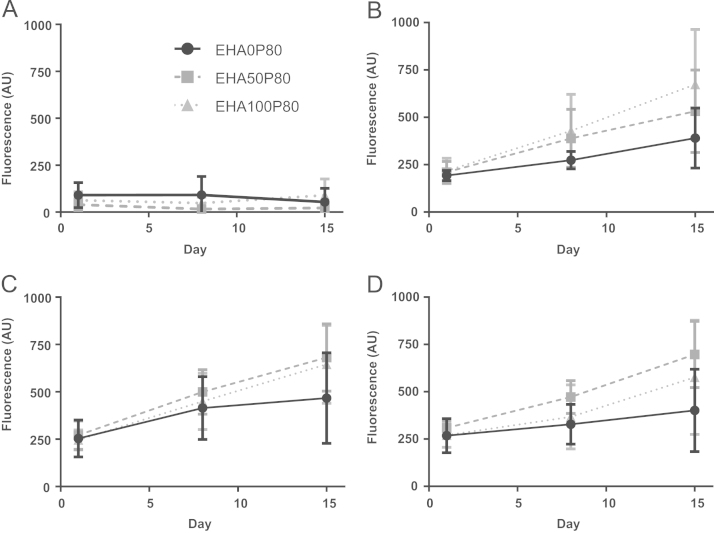
Mean±SD of resazurin fluorescence for (A) untreated (B) pcAir (C) pdAAc OIM (D) pdAAc SM samples. Untreated scaffolds did not support cell growth, pcAir and pdAAc had similar rates of increase in fluorescence intensity, with the highest proliferation occurring on compositions containing EHA. Cell viability was not affected by media composition.

Untreated scaffolds were unable to support cell attachment and growth (Fig. 5A). Plasma modification of the scaffolds clearly enhanced viable cell number on all scaffolds (Fig. 5B–D). Metabolic activity was significantly higher on plasma modified scaffolds than on untreated scaffolds for all compositions and media types on day 8 and 15 (*p*<0.05). Interestingly, there was no significant difference at any time point and composition between pcAir and pdAAc scaffolds, showing that both treatments supported similar levels of metabolic activity. Additionally, there was no significant difference observed between the two cell culture media (OIM and SM), as previously described (Delaine-Smith et al., 2012). Comparisons between scaffolds with the same plasma treatment but different wt% EHA indicated that EHA0 scaffolds supported the lowest metabolic activity, with the highest metabolic activity achieved on pcAir-treated EHA100 scaffolds and pdAAc-modified EHA50 substrates. There were no significant differences between compositions at day 8, but by day 15 significant differences with regards to composition were observed between EHA0P80 pcAir vs. EHA100P80 pcAir and EHA0P80 pdAAc SM vs. EHA50P80 pdAAc SM (*p*<0.05).

### 3.5 Confocal and two-photon microscopy

**Fig. 6:**
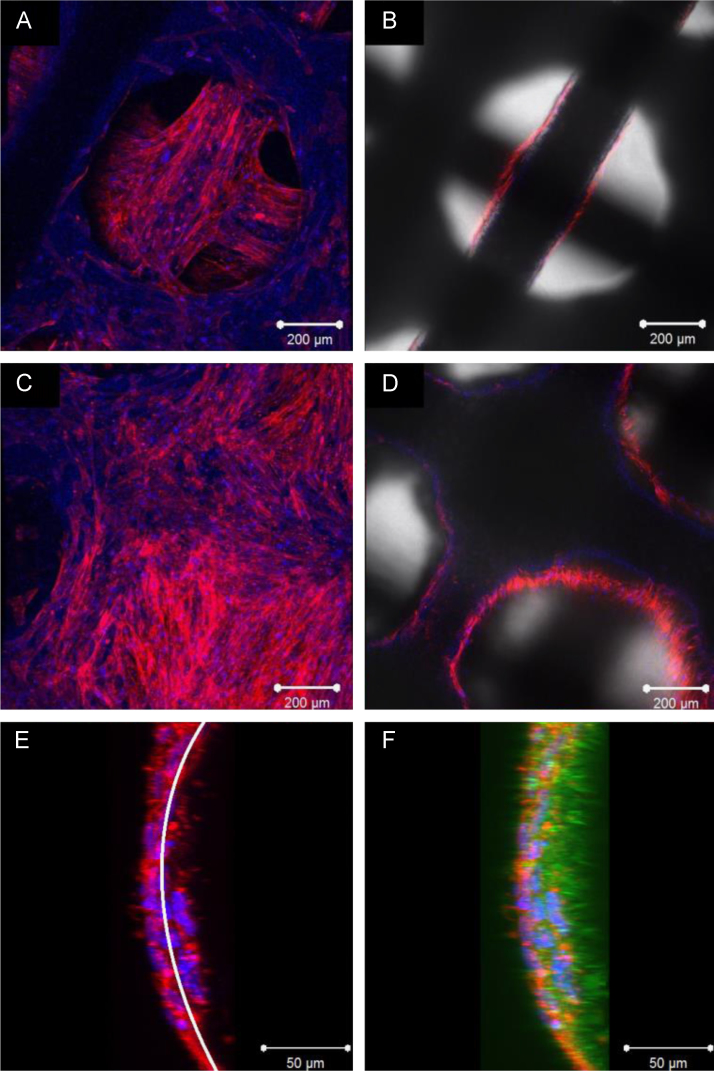
Confocal microscopy (A and C) and the corresponding DIC images (B and D) of (A) bottom two layers of a pdAAc EHA100P80 scaffold (C) the same scaffold from a different location showing the upper two layers. Note the fibre intersect is completely covered with cells and gaps between fibres were also filled. Two-photon images with (E) and without (F) the autofluorescence of the PolyHIPE (green). Fibre is viewed side on from a processed z-stack image, the curved line indicates the fibre edge. Both nuclei and actin can be seen within the fibre. (For interpretation of the references to color in this figure legend, the reader is referred to the web version of this article.)

Confocal images were taken of pcAir and pdAAc scaffolds that produced the highest RR value on day 15 (Fig. 6A–D). From the images, it can be observed that cells initially adhered and proliferated on the lowest layers indicating that the cell suspension fell to the bottom of the scaffold. On scaffolds with the highest levels of proliferation, cells were able to grow up the scaffold fibres and bridge the gaps between the fibres. Two-photon images of pcAir EHA0P80 images revealed that cells were able to penetrate the fibres (Fig. 6E and F), as shown by the presence of nuclei and actin within the material.

### 3.6 Osteogenic differentiation assay

**Fig. 7:**
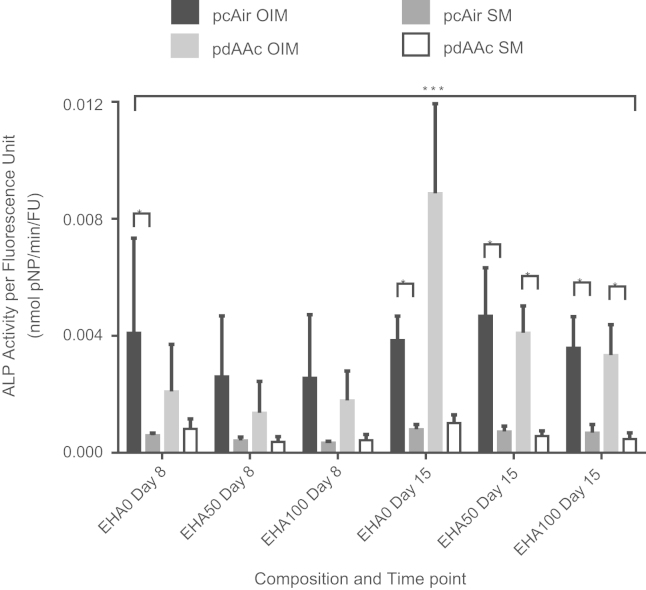
ALP activity normalised to PicoGreen fluorescence (dsDNA) present. hES-MPs cultured in OIM had higher ALP activity than their SM counterpoints at all time points (*=*p*<0.05). None of the substrates or plasma treatments were seen to significantly induce higher osteogenic differentiation, with the exception of pdAAc modified EHA0 scaffolds, which by day 15 were found to be significantly higher than all other groups (***=*p*<0.001).

In OIM, normalised ALP activity increased over time (Fig. 7). On day 8, normalised ALP activity was similar for both plasma treatments at each composition, with pcAir only being significantly higher on EHA0 scaffolds (*p*<0.05). On day 15, there was no significant difference between pcAir and pdAAc for EHA50 and EHA100 scaffolds. However, normalised activity on pdAAc EHA0 scaffolds was significantly higher than pcAir for the same composition (*p*<0.001), as well as significantly higher than both pdAAc and pcAir EHA50 and EHA100 scaffolds (*p*<0.001). Normalised activity in SM was lower than in OIM in all instances and did not increase over time. This indicates that the stiffness of the substrate did not affect osteogenic differentiation when dexamethasone was not present in the media.

## 4 Discussion

This study aimed to assess whether acrylate-based PolyHIPEs with tunable mechanical properties were suitable for bone tissue engineering. To our knowledge, this is the first time that woodpile scaffolds have been created from high internal phase emulsions using microstereolithography resulting in hierarchically porous structures for tissue engineering. A range of scaffolds was created where the fibre material was modified to give a range of mechanical properties, demonstrating that it is possible to tailor the material’s properties to the application, for example, if the application requires the scaffold to undergo load bearing or be able to accommodate a specified strain. The mechanical characterisation generated here can be used as a basic selection chart for the mechanical properties of these PolyHIPEs over a range of monomer compositions and nominal porosities. The mechanical properties describe the struts of the PolyHIPE-based woodpile structures that the cells attach to in this study. For example, a stiffness of ~30 MPa can be achieved using a composition of either EHA25P80 or EHA0P85, but the EHA25 material will undergo greater extension before failure whilst the latter has a higher UTS. A requirement of tissue engineering scaffolds is that they provide adequate mechanical support whilst new tissue is being formed, so inappropriate mechanical properties will result in a failed regeneration. For hard tissues it has been stated that a modulus in the range of 10–1500 MPa is necessary, depending on anatomical location, and for soft tissues 0.4–350 MPa (Hollister, 2005).

The surface plots show that porosity and monomer composition both affected the mechanical performance of the PolyHIPEs, but monomer composition had the greatest effect. Increasing *ϕ* from 0.75 to 0.90 reduced the stiffness by 66–75% at each monomer proportion, with the largest decrease seen between 0.75 and 0.80 *ϕ*. Increasing from 0 wt% to 100 wt% EHA decreased the stiffness by approximately 98% for all porosities, with 80–85% of this reduction seen as wt% EHA increase from 0% to 50%.

The highest UTS was achieved by an EHA25 sample as the elastomer addition allows the material to plastically deform more than the more brittle EHA0 compositions, thereby allowing it to undergo a higher tensile stress before failure. The EHA50 compositions were not able to achieve an ultimate tensile stress higher than the EHA25 composition, as at this monomer proportion the increased elasticity means that a lower force is required to achieve the same extension.

Percentage elongation at failure is not affected by nominal porosity. For the EHA0 PolyHIPEs there is no significant difference in failure strain between any of the porosities; however, by comparing the UTS of these PolyHIPEs, it can be seen that the force required to achieve the same level of extension is significantly lower as porosity increases (*p*<0.001, EHA0P75 (1.64±0.22 MPa) vs. EHA0P90 (0.51±0.10 MPa). This relationship continues across all compositions, with the UTS for the 0.90 *ϕ* composition being approximately three times lower than that for the 0.75 *ϕ* composition. This reduction in force required to achieve the same extension is due to the decrease in cross sectional area at the microscopic level, resulting in less force being required to achieve the same stress. The macroscopic cross sectional area of the 75% and 90% PolyHIPEs is still similar as tensile specimens were all cut to the same shape, hence the reduced UTS of the material but same elongation at failure. EHA100 PolyHIPEs are not the most ductile as they are too weak to undergo large extensions. Therefore, the maximum is observed where EHA and IBOA complement each other (EHA50P90, 21.86±2.87%), with the former providing sufficient ductility to allow large extensions whilst the latter strengthens the material, raising the failure stress.

The compositions selected for scaffold manufacture had the highest possible internal phase volume ratio in order to maximise the DOO whilst retaining a viscosity that is amenable to pipetting. The single-photon technique used to create the scaffolds was not capable of a smaller fibre spacing whilst retaining the fibre diameter, as partial polymerisation of the HIPE would occur between the fibres resulting in a solid sheet or web effect, depending on the distance. To remediate this in the future, a two-photon technique could be used as this permits a much higher resolution as absorption only occurs within the immediate area surrounding the focal spot. However, the single-photon technique has a manufacture time of approximately 13 min for a 13 mm×13 mm woodpile structure, producing each fibre in a single pass. A two-photon setup would take much longer, with each fibre potentially requiring multiple passes to achieve the desired width and depth. Therefore, a possible alternative would be to introduce biocompatible UV light absorbers into the continuous phase of the emulsion, reducing out-of-focal spot polymerisation and increasing resolution whilst retaining manufacture speed.

The use of PolyHIPEs in tissue engineering is still at a preliminary stage, but their capability to be used to fabricate scaffolds has begun to be assessed. It is known that the porous architecture of a scaffold can affect cell proliferation and osteogenesis (Roosa et al., 2010) and therefore optimising this will enhance the performance of the scaffold. Conventional techniques are limited in their ability to produce porous scaffolds as pore interconnectivity is often low, achieving regular and evenly dispersed porosity is limited to thin scaffolds, and it is time consuming to remove all solvents from the system (Hutmacher, 2001). PolyHIPE scaffolds produced using microstereolithography are not hampered by these problems as the porosity is formed from emulsion templating. The desired porosity is much more easily achievable and the final product will have regular, interconnected porosity throughout. This allows focus to shift onto the macroscopic structure of the scaffold, resulting in the ability to produce much more complex scaffolds. The minimum void diameter for osseous deposition is considered to be between 50 and 100 μm (Kasten et al., 2008) with the recommended size being 300 μm and larger (Karageorgiou and Kaplan, 2005). The scaffolds fibres fabricated here have pore sizes in the region of 20–30 μm, which is lower than the minimum required for bone deposition. This would be problematic if culturing on a disc of the bulk PolyHIPE material; however, the macroscopic pores formed between the fibres during the fabrication of the woodpile scaffold are between 300 μm (vertically) and 650 μm (laterally). This results in a hierarchical porosity, with a range of sizes over an order of magnitude. The presence of micro-pores creates a rougher surface topography, which increases cell attachment and may also increase cell migration (Akay et al., 2004).

It is not surprising that untreated scaffolds were unable to support cell attachment. In order to form a stable emulsion with water, the continuous phase of the HIPE must be hydrophobic and it has been clearly demonstrated that it is necessary to overcome this for a polymer to be used as a biomaterial or tissue engineering scaffold (Chang et al., 2005). To do this, two plasma treatments were selected, pcAir and pdAAc. Plasma modification is effective at penetrating the porous network of a 3-D scaffold, improving the wettability of the PolyHIPEs and consequently improving the cell adhesion (Barry et al., 2005). pdAAc adds negatively charged carboxyl groups to the surface to promote cell adhesion, and pcAir deposits oxygen-containing groups, which also have been shown to support protein and cell adhesion and improve wettability (Detomaso et al., 2005, Safinia et al., 2007). Furthermore, the inclusion of acrylic acid has been used previously to enhance cell culture on PolyHIPEs. In particular, Hayward et al. (2013b) introduced it into the internal phase of the HIPE before its inclusion into the continuous phase. After curing, they showed carboxylic functionality on the PolyHIPE pore surfaces that did not adversely affect the adhesion of human hepatocytes.

Similar cell metabolic activity on these scaffolds indicates that both treatments are suitable when improving the adhesion and proliferation on the PolyHIPEs. However, the application of pcAir is less time consuming, requires fewer processing steps (e.g. does not require liquid nitrogen to cool the monomer) and avoids handling of potentially harmful monomers (acrylic acid). Therefore, our results suggest that the simpler, faster plasma modification technique is sufficient when considering cell viability alone.

Metabolic activity on the EHA0 scaffolds made from the stiffest PolyHIPE appears to be lower than the more elastic EHA50 and EHA100 materials on both day 8 and 15. Given that fibre thickness and spacing are maintained throughout, it would be expected that relative scaffold stiffness would follow the same pattern as the material stiffness. The difference in metabolic activity between the two more elastic scaffolds is much less noticeable, which may be due to a much smaller difference in stiffness; the difference between EHA0 and EHA50 is approximately 45 MPa, whereas EHA50 to EHA100 is approximately 4.5 MPa. This lower metabolic activity on the EHA0 composition agrees with the PicoGreen data. The amount of dsDNA present is also lower on the EHA0 compositions, with little difference between EHA50 and EHA100 (data not shown). Confocal imaging demonstrated that these PolyHIPE scaffolds supported cell proliferation, and that on scaffolds with the highest levels of metabolic activity, cells groups could bridge the gaps between the fibres. However, this imaging modality cannot penetrate the scaffold fibres to investigate cell penetration. Therefore, two-photon imaging was utilised due to its ability to obtain optical sections from deeper within the sample. By exploiting the inherent autofluorescence of the material, it could be confirmed that cell ingrowth had occurred. As well as penetrating the PolyHIPE, cell processes were seen to connect through pore interconnects.

Although woodpile scaffolds formed from porous and non-porous fibres were not compared directly in this study, it is likely that differences seen when cells were grown on a microporous monolith as compared to a planar substrate are relevant to understanding the potential benefits of microporous scaffold struts. Cell ingrowth was observed into the porous PolyHIPE fibre, therefore we are confident the structure provided a 3D growth environment which would enable continuous neo-tissue formation throughout the scaffold. This is also evidenced by recent data generated in our group on microporous PolyHIPE particles (Paterson et al., in preparation). Scaffolds with non-porous fibres and macro-pores much larger than the cell size, e.g. 100s of microns, are likely to induce the same cellular behaviour as planar surfaces because the cell attaches to the strut in the same manner (shape and orientation) as a tissue culture plate (Stevens and George, 2005; Reilly and Engler, 2010). When Alvetex^®^ PolyHIPE inserts were compared to tissue culture polystyrene, it was shown that the use of these substrates profoundly improves the ability of mesenchymal stem cells to differentiate into osteogenic phenotypes. Cells retained a more physiologically relevant morphology and had increased levels of osteogenic markers, such as ALP activity, osteocalcin production, and calcium deposition (Knight et al., 2011, Reinnervate, 2015). We suggest that porous fibres will also improve diffusion-based processes throughout the scaffold.

Other groups have demonstrated the benefits of strut microporosity. For example, in selective laser sintered polycaprolactone scaffolds where a microporosity within the fibres of the scaffold was formed during the sintering process (Wiria et al., 2007, Yeong et al., 2010). The interconnected network formed was shown to improve cell ingrowth and colonisation of the scaffold. Similar to this, rapid prototyping and particulate leaching have previously been combined to introduce a controllable microporosity into scaffolds with larger macro-channels, allowing the influence of pore architecture on mechanical and biological properties to be explored (Tan et al., 2013).

Neither the composition of the scaffold nor the pdAAc coating had a significant effect on ALP activity. However, cells seeded on EHA0 scaffolds with a pdAAc coating did have significantly higher ALP activity compared to all other scaffolds, indicating this scaffold stimulated osteogenic differentiation. This suggests that the combination of the EHA0P80 PolyHIPE and pdAAc treatment resulted in the best substrate for osteogenic differentiation between those examined here. This is interesting given that EHA0 scaffolds did not result in significantly higher ALP activity than EHA50 and EHA100 scaffolds; neither did pdAAc scaffolds when compared to pcAir.

The stiffest scaffolds (EHA0P80) have a significantly lower amount of DNA (*p*<0.05) but similar metabolic activity to other scaffolds, which together with the higher ALP activity suggests that more cells in this condition differentiated rather than proliferated. It is possible that this is due to the cells response to the stiffness of the material, as substrate mechanical properties have been shown to influence stem cell fate (Engler et al., 2006, Evans et al., 2009, Tse and Engler, 2011). However, whilst stiffer substrates have been demonstrated to be conducive to osteogenic differentiation, those substrates had much lower Young’s moduli than these PolyHIPEs and cells in those previous experiments were cultured in media without dexamethasone. In addition, subsequent work suggests that stiffness alone cannot commit a stem cell to a specific lineage, with other factors such as substrate chemistry and density of cell binding ligands also influencing differentiation (Trappmann et al., 2012). For the PolyHIPEs investigated here, relative stiffness alone did not appear to induce differentiation as significantly higher ALP activity only occurs in conjunction with pdAAc. With regards to the effect of acrylic acid on osteogenic differentiation, conclusive evidence for a relationship is yet to be seen as there is evidence in the literature indicating stimulatory (Mattioli-Belmonte et al., 2005) as well as no (Seo et al., 2010) effects. It has been shown that plasma deposited acrylic acid does not diminish the cells’ ability to perceive differences in substrate stiffness when comparing the osteogenic response of MSCs to varied substrate stiffness (Colley et al., 2009). Therefore, the reason for the enhanced ALP activity could be that the pdAAc coating provides sufficient ligands for the cells to respond to the stiffer EHA0P80 scaffold fibres whereas pcAir does not. Hence, no significant difference was seen between any pcAir treatments and the stiffer scaffold material only influenced osteogenic differentiation under a specific condition.

## 5 Conclusions

To conclude, EHA/IBOA PolyHIPEs were fabricated at a range of monomer proportions and porosities demonstrating that it should be possible to predict the mechanical properties of a given composition based on its EHA/IBOA ratio. Three compositions with distinct mechanical properties were selected and used to fabricate 3-D, four layer woodpile scaffolds using single-photon microstereolithography and functionalised with either air and/or acrylic acid plasma. Compositions containing EHA were found to facilitate the highest levels of metabolic activity. Interestingly, although substrate mechanical properties alone did not significantly influence osteogenic differentiation, the stiffest scaffold material in combination with an acrylic acid treatment did induce more ALP activity in osteoprogenitor cells compared to all other conditions indicating that this substrate may be able to enhance osteogenic differentiation.

To our knowledge, this is the first time multi-layer woodpile scaffolds have been fabricated from EHA/IBOA PolyHIPEs using microstereolithography. The described fabrication method is capable of making bespoke structures from HIPEs and the use of these materials as a substrate for cell culture shows promise for tissue engineering applications where multi-scale porosities and the ability to fill a large defect are required.
